# Environmental Substances Associated with Neurodegeneration: An Overview of Parkinson’s Disease and Related Genotoxic Endpoints

**DOI:** 10.3390/genes17020236

**Published:** 2026-02-13

**Authors:** Mohammad Shoeb, Breanna Alman, Harpriya Kaur, Moon Han, Fahim Atif, William Wu Kim, Siddhi Desai, Patricia Ruiz, Gregory M. Zarus

**Affiliations:** 1 Office of Innovation and Analytics, Agency for Toxic Substances and Disease Registry, Atlanta, GA 30341, USA; 2 Office of Community Health Hazard Assessment, Agency for Toxic Substances and Disease Registry, Atlanta, GA 30341, USA; 3 Oak Ridge Institute for Science and Education, Oak Ridge Associated Universities (ORISE), 100 Orau Way, Oak Ridge, TN 37830, USA; 4 Our1World, Pine Lake, GA 30072, USA

**Keywords:** metals, Parkinson disease, biomarkers, DNA damage, neurotoxicity

## Abstract

Parkinson’s disease (PD) is a complex neurodegenerative disorder influenced by age, genetic predispositions, and environmental exposures, with a growing global incidence. This review aims to summarize findings from ATSDR Toxicological Profiles, EPA Risk Assessments, and other sources of peer-reviewed literature to examine the potential associations between PD and select metals, pesticides, and chlorinated organic compounds. Additionally, it explores using computational toxicology methods to elucidate the interactions between specific chemicals, associated genes, and their possible roles in PD. A total of 29 substances were identified to be neurotoxic with direct or probable association with PD. Risk of disease onset or symptom exacerbation of PD has been linked to exposures to neurodegenerative metals, pesticides, chlorinated organic compounds, and other environmental toxicants, alongside intrinsic factors such as genetic predisposition and aging. Supporting evidence from neurotoxicological studies directly or possibly associated with PD are summarized in referenced toxicological profiles and EPA risk assessments. Genotoxic endpoints evaluated in exposure-induced neurodegeneration including oxidative stress, DNA strand breaks, mitochondrial dysfunction, impaired DNA repair, and telomere alterations may play a critical role in linking environmental exposures to PD pathogenesis. Although these endpoints represent imperative data gaps between environmental and genetic risk factors for PD, isolating individual substances may not be necessary for prevention, as many co-occur at contaminated sites or within certain occupations. Further research is needed to clarify causal relationships between environmental exposure and genotoxic endpoints seen in neurodegenerative processes that can also be seen in PD for consideration in the development of preventive and therapeutic strategies.

## 1. Introduction

The incidence of people diagnosed with Parkinson’s disease (PD) is increasing [1,2] and is predicted to rise by approximately 76% by 2050 relative to 2021 estimates [3]. The recent estimate in North America is 47 to 77 per 100,00 among persons ages 45 and older [4]. PD is a complex neurodegenerative disorder that affects movement and includes a wide range of non-motor symptoms. It is characterized by the progressive degeneration of dopamine-producing neurons in the substantia nigra, a region of the brain responsible for coordinating movement. This dopamine deficit leads to motor function-related clinical symptoms such as tremors, rigidity, slowness of movement, and postural instability.

The diagnosis of PD also carries a non-motor function-related disease burden. These conditions can include cognitive impairment, depression, anxiety, sleep disturbance, and changes to blood pressure, digestion, and bladder control [5,6]. Although there is strong evidence supporting environmental and genetic risk factors for PD, the precise etiology of PD remains unclear; it is believed to arise from a combination of genetic and environmental factors, and biological decline due to aging [7]. Epidemiological and animal studies have demonstrated supportive evidence of an association between pesticide exposure, metal exposure [8], or solvent use and the development of PD. Likewise, genetic research has directly linked different genes to PD, primarily through case–control and Genome Wide Association Studies (GWAS). However, much of the existing literature evaluates genetic and environmental risk factors independently, and relatively few studies examine how chemicals or combinations of environmental chemicals may interact through shared biological or genotoxic pathways to influence PD risk in genetically susceptible individuals.

This review is unique because it integrates evidence from ATSDR Toxicological Profiles (TPs) and EPA risk assessments with epidemiological and experimental studies to evaluate how real-world chemicals or mixtures such as metals, pesticides, and chlorinated organic compounds may share neurotoxic and genotoxic pathways and gene–environment interactions, which may contribute to PD risk.

Environmental exposures to numerous metals and pesticides have been reported to be associated with increased risk for PD, as well as other neurological and neurodevelopmental disorders including amyotrophic lateral sclerosis (ALS), multiple sclerosis (MS), autism spectrum disorder (ASD), and attention-deficit/hyperactivity disorder (ADHD) [9,10,11,12]. Evidence from studies spanning other major neurodegenerative disorders indicates that exposure to heavy metals (e.g., lead, mercury, cadmium, manganese, aluminum) and industrial organic solvents is associated with higher odds of neurological dysfunction [9,13,14,15]. These exposure–disease relationships are further influenced by genetic variation in pathways regulating neuroinflammation, immune regulation, blood–brain barrier integrity, and xenobiotic metabolism [16]. This combined influence of environmental and genetic risk factors highlights the importance of investigating gene–environment interactions in the etiology and progression of neurodegenerative diseases such as PD. Real-world exposure scenarios often involve long-term or repeated contact with multiple environmental toxicants, making it difficult to isolate the effects of individual substances. Although important data gaps remain, focusing solely on individual exposures may not be essential for prevention, as these substances frequently occur in complex combinations. Individuals in occupational and residential settings are frequently exposed to multiple toxicants simultaneously, particularly in agriculture, manufacturing, and near hazardous waste sites [17,18]. Experimental studies demonstrate that combined exposures, such as paraquat and maneb, can produce synergistic effects on dopaminergic neurons, impersonating PD-like pathology more closely than single-agent exposures alone [19]. Similarly, co-exposure to manganese and lead has been associated with greater neurodegenerative outcomes than either metal alone [20]. These findings underscore the importance of considering cumulative and interactive exposures in both research and public health strategies [21]. Recognizing the complexity of real-world exposures is especially important when evaluating how metals, pesticides, and other environmental toxicants contribute to neurodegenerative diseases, including PD.

The Agency for Toxic Substances and Disease Registry (ATSDR) plays a role in addressing public health concerns related to exposure to hazardous substances, including those that are linked to neurodegeneration and may result in PD. As part of the U.S. Department of Health and Human Services, ATSDR conducts research and provides guidance on the health effects of toxic exposures. This includes evaluating environmental contaminants that may contribute to the initiation and development of PD. One of ATSDR’s key functions is the development of toxicological profiles, which provide comprehensive assessments of specific hazardous substances, detailing their potential health effects, exposure pathways, and risk factors. These profiles also compile information on a substance’s physicochemical properties, production, uses, health effects, toxicity, toxicokinetics, biomarkers, and minimal risk levels (MRLs), which are described as an estimation of the daily human exposure to a hazardous substance likely to pose minimal risk for non-cancer health effects. These profiles are essential for understanding how certain chemicals might influence neurological conditions like PD.

In addition to its applied research and exposure assessment functions [22], ATSDR employs computational toxicology methods to enhance its understanding of how various substances interact with biological systems at a molecular level. By utilizing advanced modeling techniques and data analysis tools, ATSDR can more efficiently predict potential health risks associated with chemical exposures. The agency also engages in surveillance activities that monitor trends in disease incidence and prevalence related to environmental exposures and can help identify populations at risk for potentially developing neurodegenerative diseases such as PD from exposure to toxic substances and inform targeted public health interventions. Furthermore, ATSDR engages in community outreach and education efforts to raise awareness about possible health risks from exposure to environmental toxins that may have the potential to affect the neurological system including the development of neurological disorders like PD. The agency collaborates with state health departments, local organizations, and other stakeholders to disseminate information about prevention strategies and available resources for affected individuals. Through these initiatives, ATSDR aims not only to protect public health but also to provide communities with knowledge about how they can mitigate risks associated with chemical exposures that may influence the onset or progression of diseases such as PD.

Given the increasing burden of PD and the growing body of evidence showing associations between environmental exposures and neurodegenerative outcomes, it is critical to better understand how specific substances and associated genotoxic endpoints may contribute to PD risk. Increasing evidence suggests that Mn accumulation in specific regions of the brain can result in neurological conditions, such as Parkinsonism [23], following welding exposure. This review summarizes findings from ATSDR Toxicological Profiles, EPA Risk Assessments, and other sources of peer-reviewed literature to examine the potential associations between PD and select metals, pesticides, and chlorinated organic compounds. The review aims (1) to utilize ATSDR’s toxicological profiles to summarize available information regarding hazardous chemical exposures, genotoxic endpoints, and neurodegeneration including PD. Additionally, (2) it explores using computational toxicology methods to elucidate the interactions between specific chemicals, associated genes, and their possible roles in PD. Many of these substances are commonly found at hazardous waste sites or are reported in occupational settings, presenting significant real-world exposure concerns. By identifying and evaluating substances with known neurotoxic and genotoxic properties, this review aims to inform public health actions, prioritize future research, and advance understanding of the potential environmental contributions to PD.

## 2. Methods

**Figure 1:**
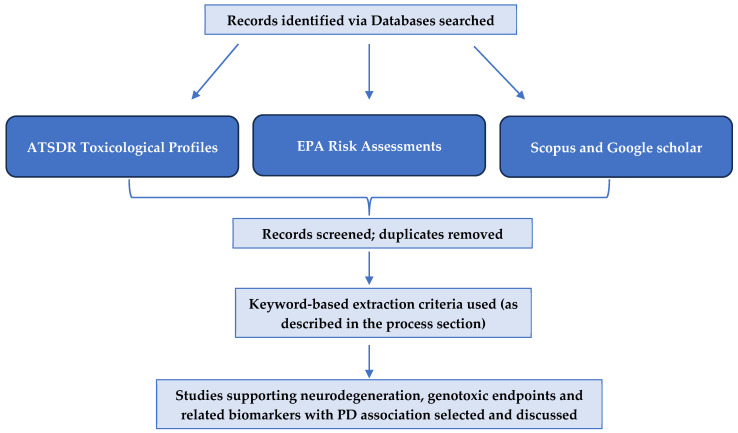
Data Collection and Screening Workflow. Note. Figure 1 outlines the methodological steps used to collect, screen, and organize data for analysis. The process includes topic selection, a literature review using ATSDR toxicological profiles and Scopus, keyword-based data extraction with inclusion/exclusion criteria, and final organization into structured tables for visualization and interpretation.

The scope of this effort focused on hazardous substances frequently encountered in the environment that fall within ATSDR’s regulatory and public health mandates. These substances are identified in ATSDR’s Substance Priority List (SPL) and are typically evaluated through ATSDR’s Toxicological Profiles or EPA Risk Assessments. Figure 1 provides a graphical summary of the methodological steps [24]. This study was based on a review of toxicological/interaction profiles and EPA Risk Assessments. This comprehensive literature served as the main source for this review article, as it pertains to both direct and indirect evidence of PD for the specific substances discussed.

Databases searched: ATSDR Tox Profiles, EPA Risk Assessments, Scopus and Google Scholar.

**Information source:** ATSDR develops publicly accessible Toxicological Profiles summarizing the health effects of toxic substances commonly found in the environment. These profiles focus on chemicals from the ATSDR’s SPL. The SPL is developed in close coordination with the Environmental Protection Agency (EPA), which compiles the National Priorities List (NPL) of hazardous waste sites prioritized for cleanup. Based on the NPL, ATSDR develops the SPL, which lists chemicals found at these sites and prioritizes them according to their toxicity, frequency of occurrence, and completed human exposure pathways. Details available at: Substance Priority List|ATSDR.

Toxicological Profiles undergo several rounds of review to incorporate emerging research. One process includes the development of a Systematic Evidence Map (SEM) report. ATSDR annually invites public nominations for new profile topics and considers special requests. Once a chemical is selected, all relevant scientific data is reviewed, and a draft document is created covering properties, use, exposure, toxicity, and biomarkers.

The draft profile undergoes peer review, and Minimal Risk Levels (MRLs) are established to assess safe exposure limits. Our TPs follow a systematic review process outlined in Appendix C of TPs ATSDR Toxicological Profiles (https://www.atsdr.cdc.gov/toxicological-profiles/site.html, accessed on 5 January 2026). After multiple expert reviews, including public comment, the final profile is published online for free access: ATSDR Toxicological Profiles.

The EPA conducts risk assessments to evaluate potential health and environmental risks from exposure to hazardous substances. They follow a structured process to inform regulatory decisions. Risk assessments focus on chemicals identified through many environmental programs, including those found at NPL sites. EPA risk assessments also undergo expert and public review. The final assessments guide regulatory actions, cleanup efforts, and safety standards for people and the environment: EPA Risk Assessment.

Process:

1. A search was conducted across the 187 ATSDR Toxicological Profiles, all associated profile addendums, and Systematic Evidence Maps (SEMs) for the term “Parkinson’s.”

The initial search identified several cited articles associated with pesticides, metals, and other chlorinated substances, including volatile organic compounds (VOCs) and semi-VOCs.

This was followed by a narrow Google Scholar and Scopus search, initiated in December 2024 and continued through March 2025, using the following search terms.

“scholarly articles on (metals, pesticides, chlorinated organic substances) associated with Parkinson’s disease”. Followed by:

“scholarly articles on (metals, pesticides, chlorinated organic substances) and (genes or genotoxic effects) associated with Parkinson’s disease”.

2. This was followed by a search of EPA Risk Assessment reports for each substance not addressed by a toxicological profile.

Selection of articles: All available studies relevant to neurodegeneration with possible associations with PD were included for each identified substance.

Three substance groups (heavy metals, pesticides, chlorinated organic substances) were reported in tables, with individual substances within each group organized by strength of evidence, ranging from strong to weak or no association.

### 2.1. Rationale and Criteria for Substance Selection

Substances included in this review were selected based on evidence reported in ATSDR TPs linking chemical exposure to neurological health outcomes commonly associated with PD, including dopaminergic neurotoxicity, Parkinsonism, motor dysfunction, and related neurodegenerative endpoints. An initial screening of all available ATSDR TPs (*n* = 187), associated profile addendums, and Systematic Evidence Maps (SEMs) was conducted using the search term “Parkinson’s.” Of the 187 profiles reviewed, 29 TPs met the inclusion criteria, as they contained epidemiological, experimental, or mechanistic evidence describing neurological outcomes relevant to PD risk. Substances lacking dedicated TPs identified through this process were further evaluated using EPA risk assessment reports. This targeted, evidence-driven approach ensured that the substances included in this review were selected based on documented neurotoxic relevance to PD rather than chemical class alone.

### 2.2. Computational Analysis

The Comparative Toxicogenomics Database (CTD) is a publicly available, manually curated resource that integrates chemical–gene–disease associations to support studies of the influence of environmental factors on human health. In this study, CTD was used to retrieve curated gene associations for environmental chemicals and PD. Overlapping gene sets for environmental chemicals were identified using Venn diagram-based tools in CTD, including VennViewer and MyGeneVenn, to visualize shared associations across chemicals and PD. The resulting gene sets were subsequently mapped onto curated PD-related pathways using the MetaCore (Clarivate, http://portal.genego.com/) pathway analysis platform. This pathway-network approach was applied to contextualize chemical–gene–disease associations within established biological processes and to support hypothesis generation regarding potential links between environmental chemical exposures and PD.

This computational analysis enables pathway-level contextualization of gene–chemical-PD associations rather than the construction of new molecular mechanisms.

## 3. Results

### 3.1. Substance-Specific Results

Several substances are associated with PD risk, including exposure to metals, pesticides, and chlorinated organic substances. The following section summarizes conclusions from toxicological profiles alongside supporting citations for each substance. Many exposures come in mixtures; thus, several of the cited references address mixtures. Animal studies permit substance-specific testing, whereas human studies often involve mixtures, especially with pesticide exposures.

### 3.2. Heavy Metals

The toxicological profiles include references for nervous system effects that potentially contribute to PD. Table 1 summarizes 43 selected studies on metals, including their exposure sources, neurotoxic mechanisms, and possible associated findings related to PD.

### 3.3. Pesticides

The toxicological profiles and many other sources include references for nervous system effects that may potentially contribute to PD risk and related disorders. Table 2 provides a summary of 50 select studies on pesticides, the sources for their exposures, mechanisms of neurotoxicity, and the findings as they pertain to PD risk. These are addressed in several toxicological profiles, risk assessments, and other referenced sources.

While several organochlorines have been found to be associated with PD [80,81,82,83,84,85,86], hexachlorobenzene had an inverse relationship in patients with other organochlorines [85]. Exposure to organochlorine insecticides appears to interact with the *ABCB1* gene to increase PD risk [84,129]. Metabolism of organophosphate is influenced by *PON1* L55M, which may decrease PON1 activity, resulting in progression of PD [105,106].

### 3.4. Chlorinated Organic Substances

Many chlorinated substances have shown to be associated with PD [132]. However, there are few recent studies that investigate genotoxic effects associated with many of them [133,134]. Table 3 summarizes key findings from 22 select studies on the above associations.

While environmental exposure studies have shown associations with findings that can also be seen in PD, genetic factors also play a critical role in disease onset and progression. A subset of PD cases is linked to mutations in genes involved in synaptic function, mitochondrial integrity, and cellular stress responses, including *SNCA*, *LRRK2*, *PARK2*, *PINK1*, and *PARK7* [154,155]. In addition to these inherited forms, growing evidence suggests that genetic susceptibility may modify the effects of environmental toxicants, contributing to inter-individual variability in risk [156]. The following summarizes key genetic and environmental factors associated with PD, including insights from toxicological profiles and literature on genotoxic, epigenetic, and molecular contributions.

### 3.5. Potential Genetic Mutations and Biomarkers

Parkinson’s disease (PD) is influenced by both genetic and environmental factors, with approximately 10% to 15% of cases linked to genetic mutations. While certain sensitive populations remain understudied, research suggests they share common genetic risk factors [157]. Several key genes play a role in PD development. One of the key characteristics of neurodegenerative pathologies is the formation of protein aggregates such as tau protein (p-Tau) and α-synuclein. Mutations in *SNCA* (Alpha-synuclein) result in abnormal protein accumulation, forming Lewy bodies, a hallmark of PD, particularly in early-onset cases [55,56,57,158]. Furthermore, accumulation of α-synuclein in PD could also occur through deposition of β-amyloid and presenilin 1 [159]. *LRRK2* (Leucine-rich repeat kinase 2) mutations are the most common genetic cause of late-onset PD, although incomplete penetrance suggests that additional environmental or epigenetic factors may influence disease manifestation [160]. The *PARK2* (*Parkin*) gene, essential for protein degradation and cellular recycling, is frequently associated with early-onset PD when mutated [161]. Similarly, mutations in *PINK1* (*PTEN-induced kinase 1*), which is crucial for mitochondrial function, contribute to early-onset PD by impairing cellular energy processes [162]. Additionally, *PARK7* (*DJ-1*) mutations reduce protection against oxidative stress, further increasing susceptibility to early-onset PD [163]. The *GBA* (*Glucocerebrosidase*) gene, which affects lysosomal enzyme activity, has also been linked to a heightened risk of PD [164]. Beyond genetic predisposition, emerging research highlights potential biomarkers of neural damage, including neurofilament light chain, sphingosine-1-phosphate, and dopamine, particularly in relation to nickel (Ni) exposure, further underscoring the interplay between genetic vulnerabilities and environmental influences in PD development [66]. Growing evidence links the genotoxicity of environmental factors to the development of neurodegenerative conditions, such as Parkinsonism [23]. Compelling evidence suggests that the elongated telomere length observed in the hippocampus of Alzheimer’s patient brains is likely due to the continuous replication of dentate gyrus cells in the hippocampus [165,166,167]. Furthermore, previous occupational exposure studies have shown alterations in PD-linked Park proteins, neuroinflammatory mediators, and glial cell activation in the brains of animal models [168]. As evidenced, this could potentially lead to persistent DNA damage, a dysfunctional shelterin complex, and the eventual development of neurodegenerative diseases, as indicated by elongated telomere length and increased expression of neurodegenerative biomarkers such as α-synuclein and presenilin 1 and 2 in brains isolated from the welding fume-exposed group [8]. Another possible contributor to neurodegeneration is the postmitotic nature of these cells, which limits their ability to divide and impairs tissue repair. Consequently, hazardous exposures may further accelerate their progression toward early cellular senescence or apoptosis, ultimately leading to neurodegenerative pathologies including PD.

These findings emphasize the complexity of PD, suggesting that both genetic and external factors contribute to disease progression, making continued research into genetic markers, epigenetic mechanisms, and environmental triggers essential for understanding and mitigating PD risk. The genetic data from these sources are summarized in Table 4.

### 3.6. Genetics Mechanisms and PD

Unlike other neurotoxicities, PD does not have a unifying cellular or molecular mechanism. Genetic mechanisms play a role in the etiology of PD, with both familial and sporadic forms identified. Mutations in several key genes, such as SNCA (which encodes alpha-synuclein), LRRK2 (leucine-rich repeat kinase 2), and PARK7 (DJ-1), have been linked to increased susceptibility to PD. These genetic alterations can disrupt cellular processes, including protein aggregation, mitochondrial function, and oxidative stress response, contributing to neuronal degeneration.

While some otherwise sensitive populations are not well studied, they appear to share some common factors [157]. Approximately 10% to 15% of PD cases are linked to genetic factors, with several key genes implicated:

### 3.7. SNCA (Alpha-Synuclein)

Mutations in SNCA lead to abnormal accumulation of alpha-synuclein, forming Lewy bodies, a pathological hallmark of PD [55,56,57,158]. These mutations are often associated with early-onset forms of the disease.

- LRRK2 (Leucine-rich repeat kinase 2): Mutations in LRRK2 are the most common genetic cause of PD in late-onset cases. However, not all carriers develop the disease, indicating incomplete penetrance [160].
- PARK2 (Parkin): The PARK2 gene, essential for protein degradation and recycling, is commonly associated with early-onset PD when mutated [161].
- PINK1 (PTEN-induced kinase 1): PINK1 mutations impair mitochondrial function and are linked to early-onset PD [162].
- PARK7 (DJ-1): Mutations in PARK7 reduce protection against oxidative stress, contributing to early-onset PD [163].
- GBA (Glucocerebrosidase): Mutations in GBA reduce lysosomal enzyme activity, increasing the risk of PD [164].
- Three serum biomarkers of neural damage (neurofilament light chain, sphingosine-1-phosphate, and dopamine) associated with Ni were involved in the development of PD [66].

### 3.8. The Gene–Environment Interaction

The gene–environment interaction for any substance depends on its physicochemical properties such as binding affinity, oxidation state, and solubility [169]. For instance, some exposures such as Pb and Hg are highly toxic [170] as compared to Ni, which is considered a weak mutagen [171]. Several of these environmental pollutants may cause minor to severe DNA damage, which is typically addressed by DNA repair machinery; however, if not repaired in a timely manner, it can lead to genomic instability and neurodegeneration [8]. Research suggests that various environmental and occupational chemical exposures contribute to genotoxic effects, potentially increasing the risk of Parkinson’s disease (PD). Below are key genotoxic endpoints associated with PD.

1. **Oxidative Stress**
  - Mechanism: Chemicals such as pesticides (e.g., paraquat, rotenone) promote the formation of reactive oxygen species (ROS), leading to oxidative DNA damage [172,173,174].
  - DNA strand breaks, 8-oxo-2′-deoxyguanosine (8-oxo-dG) formation, and oxidative base modifications [175].
  - PD Relevance: Oxidative stress damages dopaminergic neurons in the substantia nigra, a critical feature of PD pathology [176].
2. **DNA Strand Breaks**
  - Mechanism: Heavy metals (e.g., manganese, lead) and industrial solvents can induce direct or indirect DNA damage [47,177].
  - Single- and double-strand DNA breaks were detected via techniques like Comet and TUNEL assays [178].
  - PD Relevance: Persistent DNA damage in neurons is associated with neurodegenerative processes [179].
3. **Mitochondrial Dysfunction**
  - Mechanism: Mitochondrial inhibitors, such as rotenone and MPTP, cause mitochondrial DNA (mtDNA) damage [180,181].
  - Mutations, deletions, and impaired repair of mtDNA.
  - PD Relevance: Impaired mitochondrial function exacerbates neuronal vulnerability in PD [182].
4. **Epigenetic Alterations**
  - Mechanism: Organochlorines and other environmental and occupational toxicants influence DNA methylation and histone modification patterns; post-translational histone modifications, chromatin remodeling, and RNA-based mechanisms [183,184,185,186]. Notably, DDT inhibits the plasma membrane dopamine transporter (DAT) and vesicular monoamine transporter (VMAT2), further implicating its role in dopaminergic dysfunction [88,89].
  - Aberrant methylation [8] and histone changes, particularly in genes associated with neuronal health and dopamine production [185,187].
  - PD Relevance: Epigenetic dysregulation affects neuronal function and survival, contributing to PD progression.
5. **Chromosomal Abnormalities**
  - Mechanism: Chronic exposure to genotoxic agents induces chromosomal instability [188,189].
  - Aneuploidy, micronuclei formation, and chromosomal rearrangements.
  - PD Relevance: Chromosomal instability in neural progenitor cells or neurons is linked to increased neurodegeneration in PD [190].
6. **Impaired DNA Repair Mechanisms**
  - Mechanism: Certain chemicals interfere with DNA repair pathways, such as base excision repair (BER) or non-homologous end joining (NHEJ) [191]. Persistent DNA lesions and inefficient repair mechanisms [192,193,194].
  - PD Relevance: Studies show reduced DNA repair capacity in PD brains, exacerbating susceptibility to environmental insults [195].
7. **Telomere Alteration**
  - Mechanism: Telomere alteration occurs primarily due to increased telomerase activity or by dysregulated telomeric DNA-binding proteins called shelterin proteins [196].
  - PD Relevance: Telomere length alteration and epigenetic changes were observed following occupational exposure [196].

### 3.9. Genotoxicity and PD

The interplay between genetic susceptibility and environmental exposure is crucial in understanding PD. For example, individuals with PINK1 or PARK2 mutations may have heightened vulnerability to neurotoxic effects from pesticides or heavy metals. This interaction underscores the importance of identifying at-risk populations and mitigating environmental hazards.

### 3.10. Computational Toxicology Analysis

The ‘VennViewer’ function on the Comparative Toxicogenomics Database (CTD) (ctdbase.org) was used to compare genes associated with the three chemicals of interest—paraquat, lead (Pb), and trichloroethylene (TCE)—as these have strong evidence supporting an association between exposure and increased Parkinson’s disease (PD) risk. Only curated (manually reviewed) gene–chemical associations were included; inferred relationships and genes without documented interactions were excluded. The resulting gene sets were then analyzed using the MyGeneVenn tool to identify which chemically associated genes also appear in CTD’s curated PD gene–disease association dataset. In this context, the “PD” category refers specifically to genes that CTD curators have linked to PD based on available evidence. Because many genes involved in PD also participate in broader neurodegenerative or cellular stress pathways, PD-associated genes in CTD are not necessarily exclusive to PD; rather, they reflect curated evidence of relevance to PD. Each gene identified as overlapping with PD was subsequently examined individually in CTD to characterize the nature and strength of its curated relationship to PD and to determine whether the gene’s documented roles might extend to biological processes also observed in other neurodegenerative conditions.

**Figure 2:**
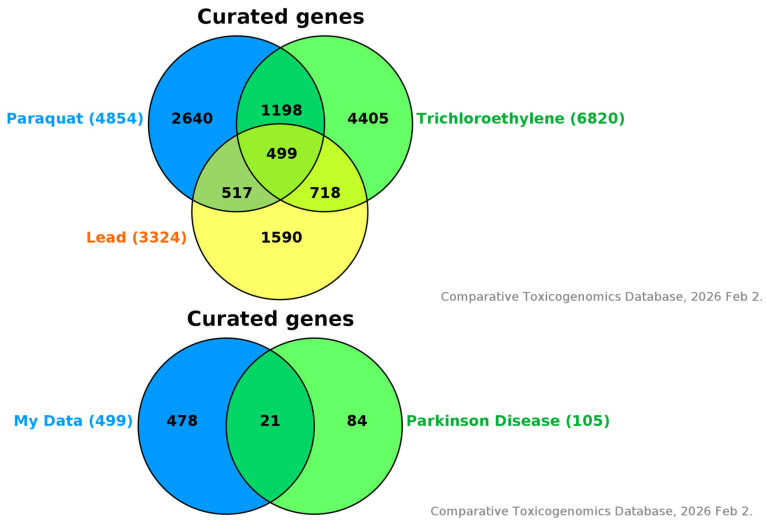
Overlap Between CTD-Curated Gene Associations for Paraquat, Lead (Pb), Trichloroethylene (TCE), and CTD-Curated Parkinson’s Disease (PD) Gene Associations. Note. Figure 2 shows two Venn diagrams. (**Top**): Venn diagram showing the number of curated gene associations for paraquat (4854), lead (3324), and trichloroethylene (6820) in the Comparative Toxicogenomics Database (CTD). A total of 583 genes are shared among all three chemicals. (**Bottom**): Of the 583 overlapping genes, 21 are also associated with Parkinson’s disease (PD), highlighting shared molecular pathways potentially involved in environmentally mediated PD risk. Data source: Data were retrieved from the Comparative Toxicogenomics Database (CTD; http://ctdbase.org), which provides manually curated chemical–gene and disease–gene associations. CTD, accessed 2 February 2026.

A total of 4854 curated gene associations with paraquat, 3324 with Pb, and 6820 with TCE were identified in the CTD as of 2 February 2026 (Figure 2). Of these 14,998 unique gene associations, 499 genes were found to be common to all three chemicals (Figure 2). Among these 499 shared genes, 21 had curated associations with PD in CTD (Table 5).

The wide range of known risk factors associated with PD suggests that it arises from complex interactions between environmental and genetic factors. Paraquat, lead (Pb), and trichloroethylene (TCE) are known to induce oxidative stress, impair mitochondrial function, and promote dopaminergic neuron degeneration—mechanisms that may increase susceptibility to neurogenerative processes seen in Parkinsonism.

**Figure 3:**
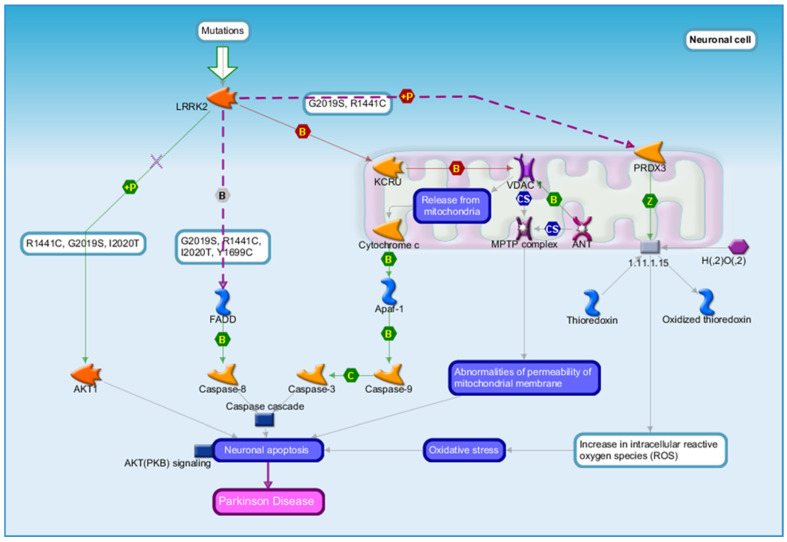
LRRK2-Mediated Pathways Contributing to PD Pathogenesis. Note. Figure 3 illustrates a MetaCore-curated LRRK2 signaling pathway. LRRK2 mutations (e.g., G2019S, R1441C, I2020T) are known to disrupt mitochondrial function, increase oxidative stress, alter vesicle trafficking, and activate downstream inflammatory and apoptotic signaling cascades. The pathway map highlights interactions between LRRK2, mitochondrial dysfunction, reactive oxygen species generation, and neuroinflammatory mediators that contribute to dopaminergic neuronal degeneration. Genes overlapping between CTD-curated chemical exposures and PD are overlaid on this pathway to illustrate how environmental toxicants may converge on established PD-relevant biological processes. Source: MetaCore (Clarivate Analytics, http://portal.genego.com/).

**Figure 4:**
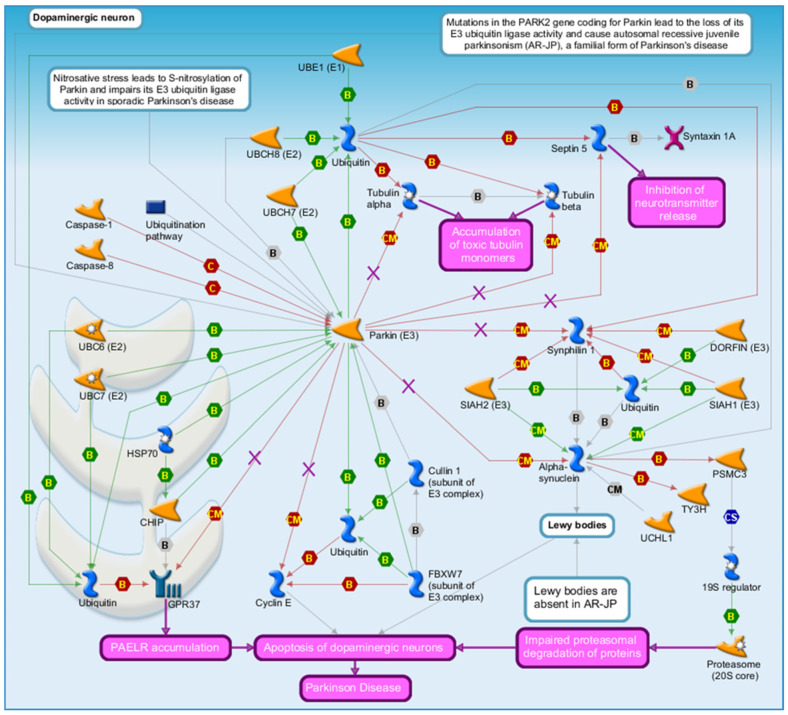
Parkin (PARK2) Pathway Dysfunction in PD. Note. Figure 4 illustrates Parkin (PARK2) pathway dysfunction in Parkinson’s disease (PD). Parkin is a key regulator of mitochondrial quality control through mitophagy and ubiquitin–proteasome-mediated protein degradation. Disruption of PARK2 signaling leads to accumulation of damaged mitochondria, impaired proteostasis, and increased neuronal vulnerability. This MetaCore-generated map illustrates how alterations in Parkin-mediated processes intersect with oxidative stress, mitochondrial damage, and apoptotic signaling. Overlaid CTD-prioritized genes provide a framework for exploring how exposure to chemicals such as TCE, Pb, and paraquat may influence these well-characterized PD pathways. Source: MetaCore (Clarivate Analytics, http://portal.genego.com/).

In this study, we use MetaCore (Clarivate) to visualize well-established Parkinson’s disease-related pathways, including LRRK2-mediated signaling (Figure 3) and PARK2 (Parkin) pathway dysfunction (Figure 4). Figure 3 and Figure 4 were generated by the authors using MetaCore-curated pathway maps and exported directly from the platform.

These pathways include key genes implicated in PD and enable pathway-level contextualization of gene–chemical associations rather than the construction of new molecular mechanisms. By leveraging MetaCore’s comprehensive database and analytical capabilities, we can better understand how alterations in these pathways may lead to the development and progression of PD, ultimately aiding in the identification of potential biomarkers and/or therapeutic targets.

The computational analysis presented here is intended to identify potential environment–gene interaction targets for further investigation and is not designed to definitively assess the strength or relevance of the relationships found in the CTD (Figure 2). Figure 3 and Figure 4 provide pathway-level context for interpreting the overlap between environmental chemical exposures and genes implicated in PD. Figure 3 illustrates the LRRK2-mediated signaling pathway, which is involved in mitochondrial function, oxidative stress, vesicle trafficking, and inflammatory responses known to contribute to PD pathogenesis. CTD-curated genes associated with trichloroethylene, lead, paraquat, and PD can be overlaid onto this pathway to visualize how environmental exposures may converge on established PD-relevant biological processes.

Figure 4 depicts the PARK2 (Parkin) pathway, a key regulator of mitochondrial quality control and proteostasis. Mapping CTD-prioritized genes onto this pathway provides a framework for exploring how chemical exposures may intersect with canonical PD pathways involving mitochondrial dysfunction and neuronal vulnerability. Together, these figures support hypothesis generation by contextualizing chemical–gene associations within well-characterized PD pathways rather than proposing new molecular mechanisms.

A key limitation of this study is that it does not evaluate the specific nature of the relationships between the chemicals and genes, or between the genes and PD. The findings demonstrate an association but do not imply causality or indicate the direction of these relationships. Additional bioinformatics, biochemical, and animal studies are necessary to more thoroughly characterize these interactions. The strength of this study lies in its novel application of a bioinformatics tool to prioritize a small set of candidate chemical–gene–disease relationships for future investigation.

This study does not propose or construct novel molecular mechanisms for trichloroethylene (TCE), lead (Pb), or paraquat. Instead, we apply a hypothesis-generating, pathway-contextual approach by overlaying CTD-curated chemical–gene associations onto previously established PD-relevant pathways. No causal or directional mechanistic inference is implied.

## 4. Conclusions

Parkinson’s disease (PD) arises from complex interactions between environmental exposures and genetic susceptibility. A total of 29 substances were identified to be neurotoxic with direct or probable association with PD. Risk of disease onset or symptom exacerbation of PD has been linked to exposures to neurodegenerative metals, pesticides, chlorinated organic compounds, and other environmental toxicants, alongside intrinsic factors such as genetic predisposition and aging. Supporting evidence from neurotoxicological studies directly or possibly associated with PD is summarized in referenced toxicological profiles and EPA risk assessments. Interpretation of these findings is limited by the reliance on ATSDR toxicological profiles and EPA risk assessments, which vary in recency and depth across substances, as well as the targeted nature of the supplemental literature searches. Evidence for several substances also remains limited or uneven in quality (not directly linked to PD), which should be considered when interpreting these findings.

Genotoxic endpoints evaluated in exposure-induced neurodegeneration including oxidative stress, DNA strand breaks, mitochondrial dysfunction, impaired DNA repair, and telomere alterations may play a critical role in linking environmental exposures to PD pathogenesis. These neurodegenerative mechanisms are further described in the genotoxicity sections of the relevant toxicological profiles.

Further research is needed to clarify causal relationships between environmental exposure and genotoxic endpoints seen in neurodegenerative processes that can also be seen in PD for consideration in the development of preventive and therapeutic strategies. Exposure to these 29 substances, possibly associated with PD, has been associated with several neurological and neurodevelopmental disorders, representing a significant health burden. Since the onset of these disorders occurs at various times throughout a lifetime, it is essential that we reduce all potential exposures at all life stages. Advances in genetic screening, such as telomere length analysis, and epidemiological research, such as DNA methylation analysis, have improved the understanding of genotoxic and neurodegenerative risk factors, paving the way for early intervention and targeted therapeutic development.

This study illustrates how computational toxicology approaches can be used to explore relationships between environmental chemicals, associated genes, and PD-relevant pathways. By integrating curated chemical–gene associations with pathway-based analyses, we provide a framework for contextualizing how chemical exposures may intersect with established PD biological pathways. These findings support hypothesis generation and prioritization for future experimental testing and epidemiological studies.

## Figures and Tables

**Table 1:** Studies supporting metal exposure-induced neurotoxic effects and possible PD associations.

Metal	Exposure Sources	Targeted Summary of Findings	References	ATSDR Minimal Risk Level (MRL)
Manganese (Mn)	Welding, mining, contaminated water, pesticides	Decreased dopamine levels in the caudate nucleus and putamen were observed, characterized by selective loss of nigral neurons projecting to the basal ganglia.	[20,25,26,27,28,29,30,31,32,33,34,35,36,37,38,39,40,41,42,43,44]	Chronic inhalation: 0.0003 mg Mn/m^3^ (0.3 µg/m^3^)
Lead (Pb)	Lead-based paints, soil, leaded gasoline, plumbing materials	Increased oxidative stress and dopaminergic neuron damage. Decreased brain volume in children, Bone Pb associated with PD.	[45,46,47,48,49]	N/A
Mercury (Hg)	Fish, dental amalgams, industrial waste	Elevated hazard ratios for diagnosis at death of PD in adults who had Hg amalgam restorations. Autopsy studies of PD patients found Hg deposited in neurons of various brain regions.	[50,51,52,53]	Chronic inhalation: 0.3 µg Hg/m^3^ (3 × 10^−4^ mg Hg/m^3^)
Copper (Cu)	Plumbing, pesticides, industrial processes	Oxidative stress and alpha-synuclein aggregation.	[54,55,56,57]	Acute oral: 0.02 mg Cu/kg/day Intermediate oral: 0.02 mg Cu/kg/day
Cadmium (Cd)	Batteries, cigarette smoke, contaminated food, fertilizers	Mitochondrial dysfunction and oxidative stress.	[58,59]	Acute inhalation: 3 × 10^−5^ mg Cd/m^3^ (0.03 µg Cd/m^3^) Chronic inhalation: 0.01 µg Cd/m^3^ Intermediate oral: 0.5 µg Cd/kg/day Chronic oral: 0.1 µg Cd/kg/day
Aluminum (Al)	Cookware, additives, antiperspirants, vaccines	Exacerbates neuronal damage.	[60,61,62,63]	Intermediate oral: 1 mg Al/kg/day Chronic oral: 1 mg Al/kg/day
Nickel (Ni)	Stainless steel, batteries, jewelry, fossil fuel emissions	Serum biomarkers of neural damage associated with Ni-induced neural damage.	[64,65,66]	Intermediate inhalation: 3 × 10^−5^ mg Ni/m^3^ Chronic inhalation: 1 × 10^−5^ mg Ni/m^3^
Arsenic (As)	Groundwater, pesticides, industrial emissions, contaminated food (e.g., rice, seafood)	Chronic exposure linked to neuronal damage and oxidative stress, with possible contributions to PD-like effects.	[67,68]	Acute oral: 0.005 mg As/kg/day Chronic oral: 0.0003 mg As/kg/day
Zinc (Zn)	Industrial activities, contaminated water, food	Dysregulated Zn homeostasis may affect alpha-synuclein aggregation and oxidative stress in PD.	[69,70]	Intermediate oral: 0.3 mg Zn/kg/day
Chromium (Cr)	Electroplating, leather tanning, contaminated water	Hexavalent chromium (Cr(VI)) exposure linked to oxidative stress and neurotoxicity, with potential relevance to PD.	[71,72]	Intermediate inhalation: 5 × 10^−6^ mg Cr(VI)/m^3^ Chronic inhalation: 5 × 10^−6^ mg Cr(VI)/m^3^ Intermediate oral: 0.005 mg Cr(VI)/kg/day Chronic oral: 0.0009 mg Cr(VI)/kg/day
Cobalt (Co)	Alloys, lithium-ion batteries, contaminated water/soil	May induce oxidative stress and neuronal damage, but evidence for direct PD link is limited.	[73,74]	Acute inhalation: 0.0003 mg Co/m^3^ Chronic inhalation: 0.0001 mg Co/m^3^ Acute oral: 0.03 mg Co/kg/day Intermediate oral: 0.02 mg Co/kg/day
Barium (Ba)	Oil/gas drilling muds, contaminated water, industrial waste	Limited evidence for direct PD risk; general neurotoxic effects could contribute to neurodegeneration.	[75]	Intermediate oral: 0.2 mg Ba/kg/day Chronic oral: 0.2 mg Ba/kg/day
Iron (Fe)	Natural in water/soil, supplements, industrial exposure	Brain Fe accumulation contributes to oxidative stress and dopaminergic cell death in PD.	[76,77]	N/A
Uranium (U)	Mining, nuclear energy, contaminated groundwater	Neurotoxicity observed, but specific links to PD are not well established.	[78,79]	Intermediate inhalation: 0.002 mg U/m^3^ Chronic inhalation: 0.0008 mg U/m^3^ Acute oral: 0.002 mg U/kg/day Intermediate oral: 0.0002 mg U/kg/day

Note. Table 1 summarizes selected metals with Minimal Risk Levels (MRLs) derived by ATSDR, representing daily human exposure estimates to hazardous substances that are likely to be without appreciable risk of adverse non-cancer health effects over a specified duration. Possible associations with PD listed here are from toxicological profiles or other scientific literature.

**Table 2:** Studies supporting pesticide exposure-induced neurotoxicity with possible PD associations.

Pesticide	Exposure Sources	Targeted Summary of Findings	References
Organochlorines: DDT, α-HCH, DDE, and dieldrin	Persistent pesticides like DDT, DDE, and dieldrin, used in agriculture	Accumulate in fatty tissues; disrupt dopamine metabolism and induce oxidative stress; linked to PD risk. While evidence remains inconsistent, studies suggest a potential increased risk in adults. Notably, DDT inhibits the plasma membrane dopamine transporter (DAT) and vesicular monoamine transporter (VMAT2), further implicating its role in dopaminergic dysfunction.	[80,81,82,83,84,85,86,87,88,89]
Glyphosate	Broad-spectrum herbicide, widely used in agriculture and gardening	Limited evidence for direct PD association; concerns about oxidative stress and mitochondrial effects. A case study linked glyphosate ingestion to Parkinson’s symptoms after four years; spatial analysis in Washington State associated glyphosate exposure with increased odds of PD-related mortality.	[90,91,92,93,94,95,96]
2,4-Dichlorophenoxyacetic Acid (2,4-D)	Herbicide used in agriculture and lawns	Evidence for neurotoxic effects but limited direct link to PD; may enhance susceptibility to other toxins. Limited human data on neurological effects of 2,4-D; epidemiological studies show no clear causal link to Parkinson’s disease, though some case–control studies suggest a potential association.	[97,98,99,100,101]
Organophosphates: chlorpyrifos and diazinon	Widely used insecticides, e.g., chlorpyrifos and diazinon	Inhibit acetylcholinesterase and may affect dopamine systems indirectly; mixed evidence for PD association.	[102,103,104,105,106]
Pyrethrin & Pyrethroids Permethrin	Common insecticides like permethrin, used in agriculture and household pest control	Affect voltage-gated sodium channels; may contribute to neuroinflammation and oxidative stress.	[107,108,109]
Atrazine	Herbicide used for weed control in agriculture and lawns	Potential to disrupt dopamine systems; indirect effects through endocrine disruption and oxidative stress.	[110,111,112,113,114,115]
Paraquat	Herbicide commonly used in agriculture	Associated with PD risk; induces oxidative stress and dopaminergic neuron degeneration.	[97,116,117,118,119]
Rotenone	Natural pesticide derived from plants; used in farming and gardening	Direct inhibitor of mitochondrial complex I; replicates PD-like symptoms in animal models.	[97,120,121]
Carbamates: ethylene-bis-dithiocarbamate, carbaryl, and aldicarb	Insecticides used in agriculture and pest control	Less persistent but linked to mitochondrial dysfunction and neuroinflammation; possible role in PD.	[17,108,122,123,124,125,126]
Fungicides: Maneb, Ziram No tox profile available	Includes maneb and ziram, often used on crops; might be measured as manganese or zinc	Linked to mitochondrial dysfunction and alpha-synuclein aggregation; synergistic effects with paraquat.	[19,88,127,128,129,130,131]

Note. Table 2 summarizes selected pesticides with neurotoxic effects and documented possible associations with PD, as cited in ATSDR toxicological profiles and other scientific literature. Minimal Risk Levels (MRLs) are due to variability in regulatory documentation and availability across pesticide classes.

**Table 3:** Studies supporting chlorinated organic compound-induced neurotoxic effects with PD associations.

Substance	Exposure Sources	Targeted Summary of Findings	References
Trichloroethylene (TCE)	Industrial solvents, contaminated groundwater	Strongly associated with increased PD risk; induces mitochondrial dysfunction, oxidative stress, and dopaminergic neuron degeneration.	[135,136,137,138,139,140]
Tetrachloroethylene (PERC)	Dry cleaning agents, contaminated drinking water	Linked to increased PD risk through oxidative stress and disruption of calcium homeostasis. Camp Lejeune studies reported a potential increased risk for Parkinson’s disease among civilian employees (SMR 2.19; 95% CI: 0.71–5.11), but the wide confidence interval indicates statistical uncertainty.	[47,141,142,143,144]
Polychlorinated biphenyls (PCBs)	Industrial coolants, contaminated fish and soil	Accumulate in fatty tissues; disrupt dopamine metabolism, induce oxidative stress, and increase neuroinflammation. Postmortem analysis of Parkinson’s disease brain tissue found significantly higher levels of PCB congener 153 and a trend toward increased total PCBs, suggesting a potential role of diorthosubstituted PCBs in PD pathogenesis.	[135,145,146]
Chloroform	Contaminated drinking water, industrial emissions	Potential contribution to oxidative damage and neurotoxicity; limited evidence for PD-specific association. No directly defined PD studies mentioned in toxicological profile.	[133,134,147]
Carbon tetrachloride (CCl_4_)	Industrial solvent, air and water contamination (before 2000 in the US)	Potential role in neurotoxicity, and PD remains under investigation. No directly defined PD studies mentioned in dated toxicological profile.	[140,148,149,150,151,152,153]

Note. Table 3 summarizes chlorinated organic compounds for which toxicological profiles and other scientific literature report associations with PD. Minimal Risk Levels (MRLs) are not included due to their absence in several relevant toxicological profiles.

**Table 4:** Genetic Mutations and Biomarkers Associated with PD.

Gene	Description	References
SNCA (Alpha-synuclein)	Mutations in *SNCA* lead to abnormal accumulation of alpha-synuclein, forming Lewy bodies, a pathological hallmark of PD. These mutations are often associated with early-onset of the disease.	[55,56,57,158]
LRRK2 (Leucine-rich repeat kinase 2)	Mutations in *LRRK2* are the most common genetic cause of PD in late-onset cases. However, not all carriers develop the disease, indicating incomplete penetrance.	[160]
PARK2 (Parkin)	The *PARK2* gene, essential for protein degradation and recycling, is commonly associated with early-onset PD when mutated.	[161]
PINK1 (PTEN-induced kinase 1)	*PINK1* mutations impair mitochondrial function and are linked to early-onset PD.	[162]
PARK7 (DJ-1)	Mutations in *PARK7* reduce protection against oxidative stress, contributing to early-onset PD.	[163]
GBA (Glucocerebrosidase)	Mutations in *GBA* reduce lysosomal enzyme activity, increasing the risk of PD.	[164]
Serum Biomarkers	Three biomarkers of neural damage—neurofilament light chain, sphingosine-1-phosphate, and dopamine—associated with nickel (Ni) exposure were implicated in PD development.	[66]

Note. Table 4 highlights key genetic mutations and emerging serum biomarkers associated with Parkinson’s disease (PD). The listed genes are implicated in early- or late-onset PD through mechanisms such as protein aggregation, mitochondrial dysfunction, oxidative stress, and impaired lysosomal function. The included biomarkers reflect neural damage linked to environmental exposures that may contribute to PD pathogenesis.

**Table 5:** PD Relevance of Shared Genes Associated with Neurogenerative Processes Across Paraquat, Lead (Pb), and Trichloroethylene (TCE) in the Comparative Toxicogenomics Database (CTD).

Gene	Function/Relevance to PD	References
ABCB1	Encodes P-glycoprotein involved in blood–brain barrier transport; altered expression may affect neurotoxicant clearance.	[197,198]
BDNF	Brain-derived neurotrophic factor; supports survival of dopaminergic neurons. Reduced levels are implicated in PD progression.	[199]
EDN1	Encodes endothelin-1, a vasoconstrictor that may influence cerebral blood flow and neuroinflammation.	[200,201]
GSTA4	Detoxification enzyme that protects against lipid peroxidation; relevant to oxidative stress seen in PD.	[202,203]
GSTM1	Glutathione S-transferase; genetic polymorphisms linked to increased PD risk, especially with pesticide exposure.	[204,205]
GSTP1	Antioxidant enzyme involved in detoxification; variants may influence susceptibility to environmental toxins.	[206]
HMOX1	Heme oxygenase-1; stress-response gene linked to oxidative stress and neuronal injury in PD.	[207,208,209]
HSPA1A	Heat shock protein involved in protein folding and cellular protection under stress conditions.	[210,211]
IGF2	Insulin-like growth factor; involved in neuroprotection and neuronal development.	[212]
IGF2R	Mediates IGF2 signaling; linked to neurotrophic and neurodegenerative pathways.	[213]
IL6	Pro-inflammatory cytokine; elevated levels are observed in PD brains and CSF.	[214,215]
KCNJ4	Potassium channel subunit; regulates neuronal excitability and may be involved in PD motor dysfunction.	[216]
LRRK2	One of the most common genetic mutations linked to PD; involved in vesicle trafficking and mitochondrial function.	[217]
MAPT	Encodes tau protein; mutations or dysregulation linked to PD and other tauopathies.	[218,219]
NQO1	Enzyme protecting against oxidative stress; polymorphisms may modify PD risk.	[220]
PINK1	Regulates mitochondrial quality control; mutations cause autosomal recessive early-onset PD.	[221]
SNCA	Encodes alpha-synuclein; central to Lewy body formation and PD pathogenesis.	[222,223,224]
SOD1	Antioxidant enzyme converting superoxide radicals; oxidative imbalance is a hallmark of PD.	[225,226]
SOD2	Mitochondrial form of SOD; important in defense against mitochondrial oxidative stress.	[227,228]
TH	Tyrosine hydroxylase; rate-limiting enzyme in dopamine synthesis, critical in PD.	[229]
TNF	Tumor necrosis factor; major inflammatory cytokine elevated in PD patients.	[230,231]

Note. Table 5 summarizes genes that are curated in the Comparative Toxicogenomics Database (CTD) as associated with all three chemicals—paraquat, lead (Pb), and trichloroethylene (TCE)—and have known or proposed relevance to Parkinson’s disease (PD). Functional descriptions include roles in oxidative stress response, neuroinflammation, detoxification, mitochondrial integrity, synaptic function, and genetic susceptibility. Gene–PD associations were derived from peer-reviewed studies and were selected based on mechanistic or clinical evidence supporting their role in PD pathogenesis.

## Data Availability

Data can be made available on request.
